# Small-Size Circulating Endothelial Microparticles in Coronary Artery Disease

**DOI:** 10.1371/journal.pone.0104528

**Published:** 2014-08-12

**Authors:** Shuai-Shuai Hu, Hong-Gang Zhang, Qiu-Ju Zhang, Rui-Juan Xiu

**Affiliations:** Institute of Microcirculation, Peking Union Medical College & Chinese Academy of Medical Sciences; Key Laboratory of Microcirculation, National Health and Family Planning Commission, Beijing, China; Brigham and Women's Hospital, Harvard Medical School, United States of America

## Abstract

**Objectives:**

Several recent lines of evidence indicate that endothelial microparticles are a new biomarker that can be used to monitor endothelial dysfunction in coronary artery disease (CAD). However, data concerning the detection of small microparticles (diameter <0.5 µm) are lacking. The aim of this study was to detect small-size endothelial microparticles (SEMPs) in CAD patients to monitor endothelial dysfunction.

**Methods:**

In total, 19 CAD patients and 14 healthy subjects were recruited. The absolute numbers and percentages of CD31^+^/CD42b^−^ SEMPs and CD62E^+^ SEMPs were determined by flow cytometry. Clinical parameters were also recorded.

**Results:**

The mean percentage of CD62E^+^ SEMPs was higher in the CAD patient group than in the healthy subject group. The area under the receiver operating characteristic curve of the percentage of CD62E^+^ SEMPs was 0.795, and the cut-off value was 1.35. There was no correlation between the percentage of CD62E^+^ SEMPs and various clinical parameters.

**Conclusion:**

The percentage of CD62E^+^ SEMPs is a potential biomarker for monitoring endothelial function in CAD.

## Introduction

By 2020, coronary artery disease (CAD) will be a major healthcare burden throughout the world [1]. Endothelial dysfunction is a key element in the development of CAD [2]. Endothelial microparticles are reportedly a new biomarker for assessing endothelial function in various diseases [3]. Indeed, numerous studies report that levels of endothelial microparticles are higher in patients with cardiovascular diseases (e.g. hypertension, CAD, and heart failure) than in healthy control subjects [4]–[6].

Endothelial microparticles are derived from endothelial cells in response to injury, activation, and apoptosis. Endothelial microparticles are around 0.1–1 µm in diameter. However, most studies have focused on those of 0.5–1 µm in diameter because conventional flow cytometry cannot provide sufficient resolution to analyze microparticles smaller than 0.5 µm (termed small–size microparticles) [7]. Many such small-size microparticles have been detected by atomic force microscopy [8]. Despite this, data concerning the detection of small-size microparticles are lacking. Therefore, it is important to study small-size microparticles in various diseases to identify new biomarkers and to develop new therapeutic approaches. It might be possible to evaluate small-size microparticles by flow cytometry by changing the signal intensity threshold. The objective of this study was to identify new biomarkers to assess endothelial dysfunction in CAD.

## Methods

### Study subjects

In total, 19 CAD patients and 14 healthy subjects were recruited. CAD was defined as ≥50% luminal diameter stenosis of a major coronary artery, including the left main coronary artery, left anterior descending artery, left circumflex artery, and right coronary artery by coronary arteriography. Patients with a history of chronic renal failure requiring dialysis, hepatic or hematologic disorders, or inflammation, autoimmune, or malignant diseases were excluded. Healthy subjects were included if they had no known history of medical illness, normal blood pressure (<140/90 mmHg), and appeared healthy in a physical examination. The protocol regarding this study was approved by the Ethics committee at institute of Microcirculation Peking Union Medical College & Chinese Academy of Medical Sciences, and verbal informed consent was received from each study subject before entering the study.We got verbal informed consent because patients and healthy subjects told us blooding drawing was a kind of noninvasive test.So it was not necessary to write informed consent.Before drawing blood of every experiment subject,we told them the purpose of drawing blood, we also paid some money for the subjects. So the experiment subjects were willing to attend our experiment and gave verbal informed consent. The ethic committee approved this consent procedure. Clinical and laboratory data were collected from all subjects.

### Isolation and measurement of small-size endothelial microparticles (SEMPs)

Microparticles were isolated using a single protocol to avoid variations in pre-analytical procedures. Blood samples were obtained by venipuncture and transferred to blue-top vacutainer tubes containing sodium citrate. Within 1 hour of sampling, whole blood samples (3 ml each sample) were centrifuged at 1500 g for 10 min to prepare platelet-rich plasma and then centrifuged again at 13,000 g for 10 min to obtain platelet- free plasma. Thereafter, samples were stored at −20°C for 1 week and then at −80°C until analyzed. Microparticle levels were not affected by thawing the plasma stocks once. For the SEMPs assay, platelet-poor plasma (50 µl) was incubated with both of the following fluorescent monoclonal antibodies (4 µl each): phycoerythrin (PE)-labeled anti-CD31 (catalogue number 560983, BD Biosciences) and PE-labeled anti-CD62E (catalogue number 551145, BD Biosciences). Thereafter, the samples were incubated at room temperature for 20 min, diluted with 1 ml of phosphate-buffered saline, and analyzed by flow cytometry (Accuri C6, Accuri Cytometers). For specific delineation of CD31^+^ endothelial microparticles, and not of platelet-derived CD31^+^ microparticles, CD42b^−^ microparticles were analyzed in platelet-free plasma. For flow cytometry analysis of SEMPs, a signal intensity threshold of 35,000 was used and standard beads (0.46 µm diameter; Sigma, St. Louis, MO) were used for calibration. Forward scatter intensity versus side scatter intensity dot plots were generated by gating on microparticles, followed by one- or two-color fluorescence histograms. Accuri C6 software was used to quantify the absolute numbers of SEMPs per unit of sample. In all measurements, an isotype control antibody was used as the negative control. SEMPs were defined as CD31^+^/CD42^−^ or CD62E^+^. Values are reported as counts per µl of peripheral blood. The laboratory personnel who performed these assays were blinded to all clinical data and the study participants.

### Statistical analysis

Data are expressed as the mean ± standard deviation (SD) or as numbers. Variables were evaluated using the Kolmogorov-Smirnov test to assess normality. All data shown were normally distributed. Differences between groups were analyzed using the Student's test (two-sided). The chi-square test was used to compare quantitative and categorical variables. Multiple linear regression analysis was used to determine whether various factors were independently associated with the percentage of CD62E^+^ SEMPs. The ability of the percentage of CD62E^+^ SEMPs to predict CAD was determined using a receiver operating characteristic (ROC) curve. The area under the ROC curve (AUC, 95% confidence interval (CI)) measured the accuracy of the diagnostic test. Values close to 0.5 indicated failure of the test, whereas values close to 1 indicated that the accuracy of the test was virtually perfect. The cut-off value for the percentage of CD62E^+^ SEMPs was determined using Youden's test. A value of P<0.05 was considered significant. Statistical analyses were performed using SPSS 17.0.

## Results

### Clinical characteristics and SEMPs analysis of study subjects

**Figure 1:**
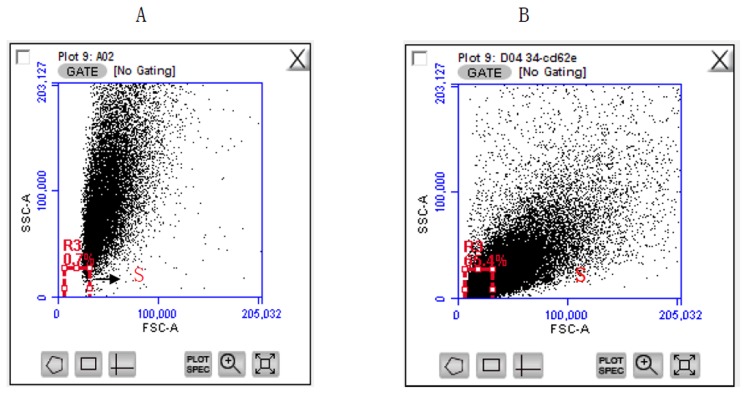
Representive flow cytometric data of circulating SEMPs. (A) Side scatter intensity versus forward scatter intensity dot plot derived from a sample containing standard beads (0.46 µm diameter). (B) Side scatter intensity versus forward scatter intensity dot plot derived from a blood sample containing SEMPs, which were defined as those with a diameter of ≤0.46 µm. S window was a gate of MP size.

**Figure 2:**
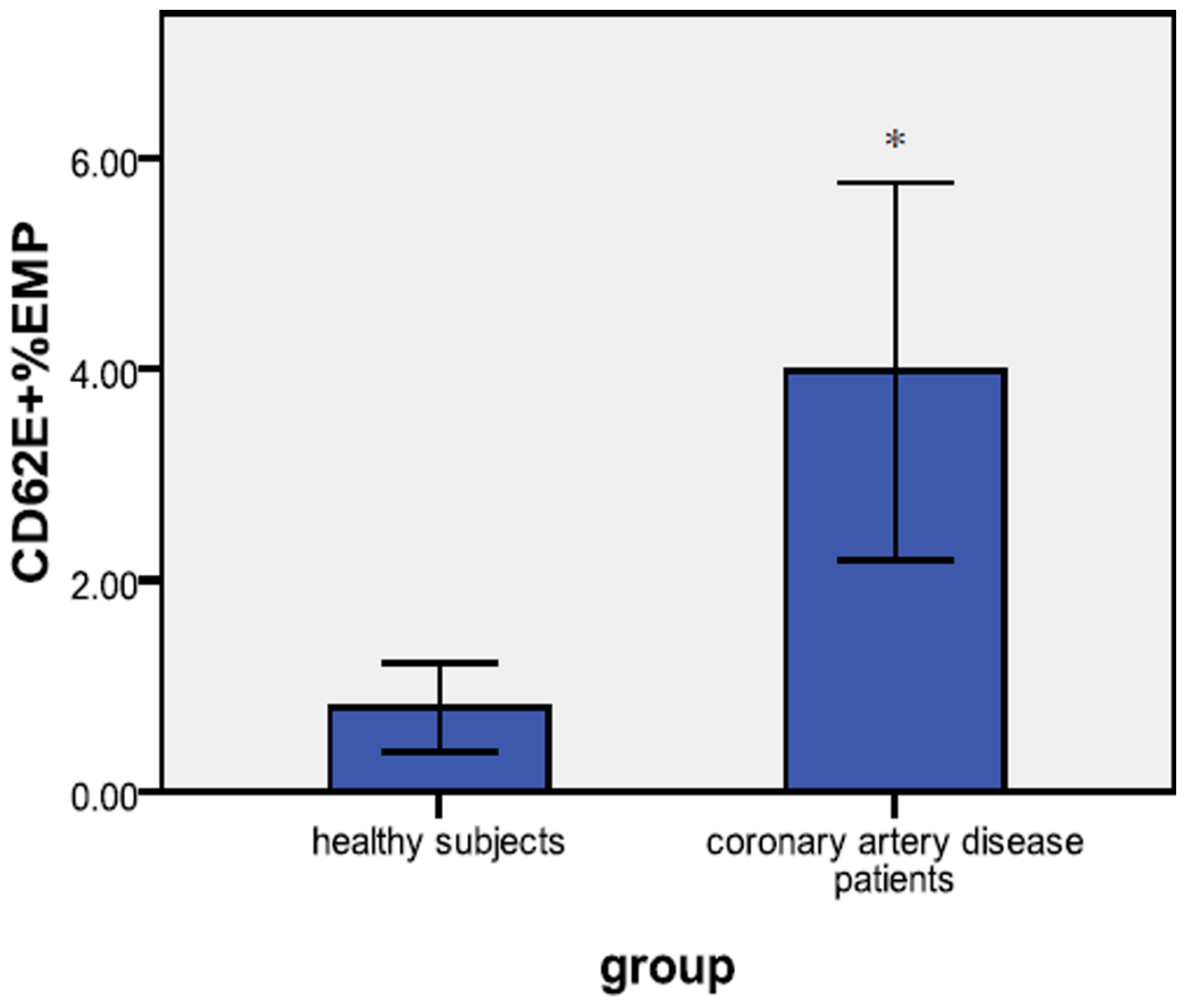
Percentage of CD62E^+^SEMPs in CAD patients and healthy subjects. The graph shows the mean percentage of CD62E^+^ SEMPs in blood samples obtained from CAD patients and healthy subjects (mean ± SD). *P<0.05.

Representative plot of Flow cytometry-analysis was shown in Figure 1. The mean percentage of CD62E^+^ SEMPs was markedly higher in CAD patients than in healthy subjects (p = 0.004), meanwhile, the number of CD31^+^EMP showed no difference between CAD patients and healthy subjects, the results were shown in Table 1 and Fig. 2. The mean age of the healthy subjects was less than that of the CAD patients (p<0.001), the mean body mass index of the CAD patients was higher than that of the healthy subjects (p = 0.003), and the mean level of low-density lipoprotein in healthy subjects was lower than that in CAD patients (p = 0.031), the proportion of men was higher in the CAD patient group than in the healthy subject group (p = 0.013), the results were shown in Table 2.

**Table 1:** Characters of SMPs in healthy subjects and coronary artery disease patients.

Characteristic	Healthy controls	Coronary artery disease patients	p-value
**CD31^+^/CD42b^−^ SEMPs**	78.28±109.74	112.18±186.25	0.608
**Percentage of CD31^+^/CD42b^−^ SEMPs**	2.47±4.56	9.08±15.80	0.219
**CD62E^+^ SEMPs**	20.48±18.34	42.63±82.94	0.443
**Percentage of CD62E^+^ SEMPs**	0.87±0.57	3.24±2.37	0.004

SEMP: small-size endothelial microparticles A value of P<0.05 was considered significant difference.

**Table 2:** Clinical data of healthy subjects and coronary artery disease patients.

Characteristic	Healthy controls	Coronary artery disease patients	p-value
**Age, years**	33.86±6.32	68.47±14.15	<0.001
**Gender (female/male)**	11/3	6/13	0.013
**BMI**	22.42±1.65	25.53±3.37	0.003
**TC concentration, mmol/l**	4.00±0.57	4.32±0.84	0.222
**HDL concentration,mmol/l**	1.34±0.95	1.23±0.24	0.082
**LDL concentration,mmol/l**	2.18±0.53	2.83±1.09	0.031
**TG concentration,mmol/l**	1.50±0.48	2.11±1.33	0.078
**DM**	0(10)	4(19)	0.119
**Medication**			
**Platelet inhibitor**	0(10)	10(19)	-
**Beta-blocker**	0(10)	3(19)	-
**ACE-I/ARB**	0(10)	2(19)	-
**Calcium channel blockers**	0(10)	5(19)	-

Values are expressed as the mean ±SD or as the number of subjects. BMI: body mass index; DM: diabetes mellitus; TC: total cholesterol; HDL: high-density lipoprotein; LDL: low-density lipoprotein; TG: triglyceride; ACE-I/ARB: angiotensin-converting enzyme inhibitor/angiotensin II receptor blocker. A value of P<0.05 was considered significant difference.

### Correlations between the percentage of CD62E^+^ SEMPs and clinical parameters

Multiple linear regressions were performed. Age, gender, body mass index, total cholesterol concentration, diabetes mellitus, high-density lipoprotein concentration, low-density lipoprotein concentration and triglyceride concentration were used as independent variables, and the percentage of CD62E^+^ SEMPs was used as the dependent variable. The percentage of CD62E^+^ SEMPs was not correlated with any of these clinical parameters.

### Usefulness and Accuracy of CD62E^+^SEMP% as a monitor endothelial dysfunction factor in CAD

**Figure 3:**
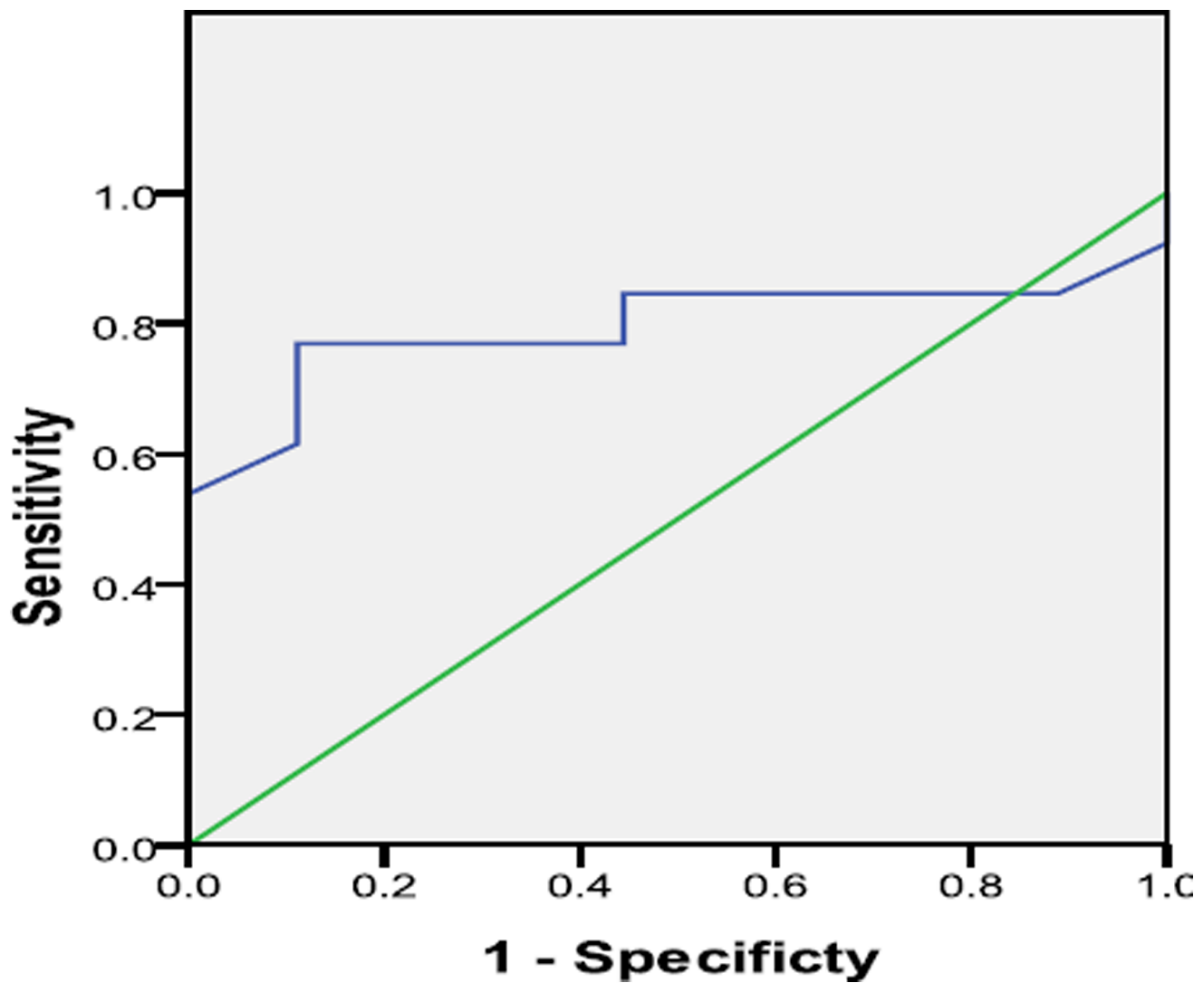
ROC curve of the percentage of CD62E^+^ SEMPs. The area under the curve of the percentage of CD62E^+^ SEMPs was 0.795.

To evaluate the ability of CD62E^+^SEMPs to monitor endothelial dysfunction in CAD, a ROC curve was constructed and shown in Fig. 3. The area under the ROC curve was 0.795 (95% CI,0.595–0.995, P = 0.021). The optimal cutoff value of CD62ESEMP obtained from the Youden index was 1.35%, with sensitivity, specificity values of 76.9% and 88.9%, respectively.

## Discussions

ROC curve for CD62E^+^ SEMPs was analyzed and the cut off value of 1.35% had a sensitivity of 76.9% and specificity of 88.9%. According to the value of AUC (0.795), CD62E^+^SEMPs showed a high accuracy to monitor endothelial dysfunction. Therefore, Our study is the first time to show CD62E^+^SEMPs could be a new informative biomarker to monitor endothelial dysfunction in CAD.

The presence of CD62E^+^ endothelial microparticles is an indication of endothelial activation [9]; therefore, the results of the present study suggested that endothelial activation participates in the pathogenesis of CAD. The percentage of CD62E^+^ SEMPs is likely to be a biomarker for endothelial function in CAD.

Due to the limitations of flow cytometry, most previous studies focused on endothelial microparticles (diameter ≥0.5 µm) larger than those examined in the present study. The current study specifically focused on small-size microparticles and thus is of particular interest and importance. The new study method designed for the evaluation of small events allows analysis of undetected majority endothelial microparticles, using below 0.46 um polystyrene beads in size,termed small-size SEMP(<0.5um) [9]. Consequently, caution should be exercised when comparing the results of the current study with those of previous studies. The present study suggests that the percentage of CD62E^+^ SEMPs, rather than the absolute numbers of CD62E^+^ SEMPs, is an indicator of endothelial function in CAD. In addition, this study demonstrated that the number and percentage of CD31^+^/CD42b^−^ SEMPs, an indicator of endothelial cell apoptosis [10], showed no difference between CAD patients and healthy subjects. This further indicates that apoptosis of endothelial cells may does not play a key role in the pathogenesis of CAD.

According to correlation and regression analysis, the percentage of CD62E^+^ SEMPs did not correlate with age, gender, body mass index, diabetes mellitus, high-density lipoprotein concentration, low-density lipoprotein concentration, triglyceride concentration, or various other clinical parameters. Furthermore, this analysis showed that the percentage of CD62E^+^ SEMPs is a important biomarker for assessing endothelial function in CAD.

CD62E molecular belongs to the selectin family of adhesion molecules and its expression is related to inflammation, endothelial dysfunction, and coagulation [11]. However, the mechanisms that link CD62E to these processes need to be further elucidated.

This study has limitations. First, the numbers of CAD patients and healthy subjects recruited were relatively low. Second, we did not determine whether analysis of CD62E^+^ SEMPs can be used for the prognosis of CAD patients. This needs to be investigated in future studies.

## Conclusions

The results of this study indicate that the percentage of CD62E^+^ SEMPs can be used to monitor endothelial function in CAD. In ROC curve analysis, the cut-off value for the percentage of CD62E^+^ SEMPs was 1.35. Further studies are needed to determine the mechanism linking CD62E^+^ SEMPs with endothelial function in CAD.
