# Correction: Small-Size Circulating Endothelial Microparticles in Coronary Artery Disease

**DOI:** 10.1371/journal.pone.0111994

**Published:** 2014-10-22

**Authors:** The PLOS ONE Staff

The grant number for the National Natural Science Foundation of China is incorrect. Please refer to the complete funding statement here:

This study is supported by the National Natural Science Foundation of China (Grant No. 11274046) & Peking Union Medical College Innovation Funding (No. 3332013021). The funders had no role in study design, data collection and analysis, decision to publish, or preparation of the manuscript.
